# Changes in miRNA expression and retinal blood vessels are associated with short-term air pollution exposure.

**DOI:** 10.1186/2049-3258-73-S1-P24

**Published:** 2015-09-17

**Authors:** Tijs Louwies, Luc Int Panis, Patrick De Boever, Tim Nawrot

**Affiliations:** 1 Hasselt University, Diepenbeek, Belgium; 2 Flemish Institute for Technological Research (VITO), Mol, Belgium

## 

Air pollution, a cardiovascular risk factor, might exert its effects through the microcirculation. Fundus photography allows study of the retinal vasculature in vivo. Short-term PM_10_ exposure is associated with changes in retinal blood vessels, but the underlying mechanism remains unknown. Expression of MIR21, MIR222 and MIR146A, gene regulators involved in oxidative stress and inflammatory processes, can be changed by air pollution and might be a pathway explaining the association between PM_10_ and microvascular changes.

50 healthy adults (50% women, 50% men, 23-58 years old) were sampled once a month from December 2014 until April 2015. At each study visit fundus photos and venous blood samples were collected. PM_10_ data were obtained from a nearby monitoring station. Image analysis was used to calculate the width of retinal blood vessels, represented as the Central Retinal Arteriolar/Venular Equivalent (CRAE/CRVE). miRNA was isolated from blood and expression was measured using qRT-PCR. Mixed models were used for statistical analysis.

Short-term changes in PM_10_ exposure were associated with changes in CRAE, CRVE and miRNA-expression. Each 10 μg/m^3^ increase in PM_10_ during the previous 24 hours was associated with a 0.58 μm decrease (95% CI: -1.16, -0.0005; p=0.056) in CRAE, a 0.99 μm increase (95% CI: 0.18, 1.80; p=0.021) in CRVE, a 6.6% decrease (95% CI: -11.07, -2.17; p=0.0038) in miR-21 expression and a 6.7% decrease (95% CI: -10.70, -2.75; p=0.0012) in miR-222 expression. miRNA expression was associated with CRAE and CRVE. Each 10% increase in miR-21 expression and miR-222 was associated with respectively a 0.14 μm increase (95% CI: 0.0060, 0.24; p=0.046) in CRAE and a 0.28 μm decrease (95% CI: -0.50, -0.062; p=0.016) in CRVE.

PM_10_ exposure affects miRNAs involved in inflammation and oxidative stress. These changes may be an underlying mechanism for the association between PM_10_ exposure and retinal arteriolar narrowing and venular widening.

